# Facing Loneliness and Anxiety During the COVID-19 Isolation: The Role of Excessive Social Media Use in a Sample of Italian Adults

**DOI:** 10.3389/fpsyt.2020.586222

**Published:** 2020-12-08

**Authors:** Valentina Boursier, Francesca Gioia, Alessandro Musetti, Adriano Schimmenti

**Affiliations:** ^1^Department of Humanities, University of Naples Federico II, Naples, Italy; ^2^Department of Humanities, Social Sciences and Cultural Industries, University of Parma, Parma, Italy; ^3^Kore University of Enna, Faculty of Human and Social Sciences, Enna, Italy

**Keywords:** anxiety, isolation, loneliness, excessive social media use, COVID-19

## Abstract

The outbreak of coronavirus disease 2019 (COVID-19) prompted people to face a distressing and unexpected situation. Uncertainty and social distancing changed people's behaviors, impacting on their feelings, daily habits, and social relationships, which are core elements in human well-being. In particular, restrictions due to the quarantine increased feelings of loneliness and anxiety. Within this context, the use of digital technologies has been recommended to relieve stress and anxiety and to decrease loneliness, even though the overall effects of social media consumption during pandemics still need to be carefully addressed. In this regard, social media use evidence risk and opportunities. In fact, according to a compensatory model of Internet-related activities, the online environment may be used to alleviate negative feelings caused by distressing life circumstances, despite potentially leading to negative outcomes. The present study examined whether individuals who were experiencing high levels of loneliness during the forced isolation for COVID-19 pandemic were more prone to feel anxious, and whether their sense of loneliness prompted excessive social media use. Moreover, the potentially mediating effect of excessive social media use in the relationship between perceived loneliness and anxiety was tested. A sample of 715 adults (71.5% women) aged between 18 and 72 years old took part in an online survey during the period of lockdown in Italy. The survey included self-report measures to assess perceived sense of loneliness, excessive use of social media, and anxiety. Participants reported that they spent more hours/day on social media during the pandemic than before the pandemic. We found evidence that perceived feelings of loneliness predicted both excessive social media use and anxiety, with excessive social media use also increasing anxiety levels. These findings suggest that isolation probably reinforced the individuals' sense of loneliness, strengthening the need to be part of virtual communities. However, the facilitated and prolonged access to social media during the COVID-19 pandemic risked to further increase anxiety, generating a vicious cycle that in some cases may require clinical attention.

## Introduction

The outbreak of coronavirus disease 2019 (COVID-19) generated a global health crisis, prompting people to face a distressing and unexpected situation. The risk of contamination and the experience of social distancing changed people's behaviors and deeply impacted individual feelings, daily habits, and relationships. Uncertainty about the timeline of the growing pandemic strengthened people's fears (1–3), stress, and confusion (4). Isolation and restrictions due to quarantine worsened feelings of anxiety and loneliness among both older and younger populations (1, 5). Since the first weeks of COVID-19 diffusion, scholars worldwide have started to investigate how the pandemic has been impacting mental health (6–12) and has been forcing individuals to cope strategically with their isolation (13). Indeed, the loss of one's usual routine and reduced social contacts may cause boredom, frustration, and a sense of isolation, which can generate high levels of distress in individuals increasing the risk of mental disorders, such as anxiety, mood, addictive, and thought disorders (14–21). In this regard, a strong participation of mental health professionals in the management of the crisis and post-crisis has been warmly recommended, in order to help people facing the stressful circumstance and its risky consequences (6, 20).

The subjective sense of loneliness describes individuals' disagreeable feeling of having a lack of meaningful social relationships (22–24), concerning both quantity and quality of social contacts (25, 26). Even though the subjective feeling of being lonely does not overlap with objective social isolation (24, 27), social isolation is undoubtedly one of the strongest predictors of loneliness and has negative effects on both health and well-being (28). Indeed, social inaction and resultant isolation frequently worsen individuals' sense of loneliness (29).

Several studies have examined sociodemographic and contextual variables related to loneliness, such as age and social status, highlighting that different life circumstances are meaningfully associated with loneliness (30–36). In particular, difficult life conditions and drastic changes in social contexts have been linked to increased social and emotional loneliness (37–39). In any case, loneliness seems to reflect an individual's unsatisfied desire to enjoy close contacts with people and to be embedded in significant relationships; thus, it represents an individual's failure in social domains of life that play a key role in human well-being (24). Indeed, perceived feelings of loneliness have been reported as a specific risk factor for anxiety and chronic stress (24, 40, 41), as well as for high engagement in unhealthy behaviors (10, 42). Additionally, social isolation and loneliness have been positively associated with anxiety in both older adults and youth (29, 43), and negatively associated with happiness, well-being, and life satisfaction (44–46). However, some research also suggests that individuals with high emotional loneliness are more prone to engage in adaptive coping behaviors, such as creating new social interactions (47).

Social relationships are core elements in people's lives. Thus, how individuals cope with loneliness during forced isolation is important to the debate (48, 49). Within this context, the use of digital technologies, and particularly social media, may serve connective functions in helping individuals to increase their social capital (50, 51). Social media refers to producing, receiving, and sharing online content, including a wide range of Internet-related communication and social applications, such as online virtual games, blogs, e-health forums, and social networking sites.

Social restrictions during the COVID-19 pandemic have forced individuals to face a potentially terrifying reality of isolation (1); thus, people worldwide have been invited to be socially (but not physically) connected (52). Even the American Psychological Association (APA) has promoted connections via social media platforms for safety and to be informed and relieve stress during the COVID-19 pandemic (53). Indeed, social media can play a key positive role in communication by allowing people to feel that they are not alone but part of a community (1). Additionally, social media have been proposed as tools for alleviating anxiety among individuals, even though the specific effects of social media consumption need to be carefully addressed (54). For instance, analytics company Sprinklr reported nearly 20,000,000 people mentioning coronavirus-related terms on social media in the last months (55), highlighting the heavy role of COVID-19 on people's cognition and behaviors. Within this context, the World Health Organization (WHO) reported about the risks of the “infodemic” causing information overload and widespread anxiety during the pandemic (56, 57) and thus recommended calling on official information sites to avoid excess or incorrect information (58). Likewise, the risk of a “digital epidemic” has been evidenced (59), which must be stemmed by using technology creatively (54) and by moderating media exposure and consumption, to prevent people from becoming overwhelmed (53).

In this regard, social media use highlights clear—yet risky—opportunities (60), depending on its specific use or misuse, for individuals to face isolation through social connection and quench their own thirst for knowledge and communication. Indeed, excessive media consumption and steady health messaging on COVID-19's diffusion and consequences are exacerbating factors on individuals' mental health (8). Previously, negative feelings have been extensively associated with excessive social media use and digital addiction (61–70). In particular, loneliness is a risk factor related to problematic engagement in Internet-related activities (63, 71–75) and as one of the most important predictors of problematic Internet and social networking site use (76, 77). In fact, lonely individuals may see the online environment as an ideal place for increasing their opportunities for interaction and belonging (78, 79), but this use may sometimes become maladaptive and excessive.

In fact, a worrisome and vicious cycle between loneliness and excessive Internet use has been evidenced (80–84), with a bidirectional (i.e., reciprocal) relationship especially observed between loneliness and problematic social networking site use, particularly in late adolescents and adults (85). In sharp contrast, other studies have demonstrated that social media use may help people to decrease their sense of loneliness (86) while increasing their perceived social support, self-esteem, happiness, and satisfaction (87, 88). For instance, Internet use for entertainment, online communication, and social interactions may serve adolescents' and young adults' need to face their loneliness (89, 90). Also, Sum et al. (91) reported that higher levels of emotional loneliness were associated with greater use of the Internet for social connections.

Thus, mutual connections between loneliness and individuals' responses to the COVID-19 outbreak should be better understood (8). People's levels of engagement in social media during the pandemic likely deserve attention because these levels might reflect adaptive or maladaptive responses to the distressing situation. Indeed, individuals could be highly involved in social media as a strategy to cope with their sense of loneliness, thus revealing their need to be connected with other individuals and to alleviate their negative mood. In this context, individuals' use of the online environment may alleviate negative feelings caused by distressing life circumstances, while potentially leading to problematic use and addictive-like symptoms (92). Indeed, within a compensatory model of problematic Internet-related activities, reactions to negative life circumstances are facilitated by Internet applications, which might lead to both positive (e.g., alleviating negative feelings or fulfilling the need for social contacts) and negative (e.g., reinforcing problematic engagement) outcomes (92).

### The Present Study

In light of the theoretical premises and the research evidence, we hypothesized that individuals experiencing high levels of loneliness during the COVID-19 pandemic were more prone to experiencing feelings of anxiety and to be dysfunctionally involved in social media use, probably as a strategy to cope with their sense of loneliness. Consequently, we explored whether individuals experiencing high levels of loneliness during the forced isolation for COVID-19 were more prone to feel anxious and whether their sense of loneliness prompted excessive, addictive-like, use of social media. Furthermore, the mediating effect of excessive social media in the relationship between perceived loneliness and anxiety was tested.

## Materials and Methods

### Participants and Procedure

A total of 715 adults responded to an online survey during the period of pandemic lockdown for COVID-19 in Italy (from the 1st to the 30th of April 2020). The sample comprised 204 men (28.5%) and 511 women (71.5%) aged between 18 and 72 years, with a mean age of 31.70 years (*SD* = 10.81). Participants were recruited through advertisements in Internet communities of Italian University students and other online groups (via social media platforms), and the groups' members were asked for dissemination in their turn. Therefore, a snowball sampling method was adopted as a recruitment strategy. The call for participation in the online study contained a website link for participants to click on to complete the questionnaire. Before filling out the survey, all of the participants were informed about the research aims and scopes and the measures to be used in generating the data. Participation was voluntary, and confidentiality and anonymity were assured. The participants could withdraw from the study at any time. No course credits or remunerative rewards were given. The study was approved by the University Federico II (Naples, Italy). Research Ethics Committee and was conducted according to the ethical guidelines for psychological research established by the Italian Psychological Association (AIP).

### Measures

#### Sociodemographic Information and Social Media Use Patterns

In this section, information was collected about gender, age, marital status, whether the participant was living alone during the quarantine, the most used social media, and hours per day spent on social media before and during forced isolation due to COVID-19.

#### Italian Loneliness Scale

The Italian Loneliness Scale (ILS) (24) is a 20-item self-report scale rated on a 4-point Likert scale (from 1 = *never* to 4 = *always*) that evaluates perceived loneliness. Eighteen items were constructed by adapting items from the widely used University of California Loneliness Scale (23) and the Dutch De Jong Gierveld Loneliness Scale (93). Additionally, two single-item criterion measures were derived *ad hoc* for the Italian scale, referring to a brief time interval (7 days), and were the last two items in the scale (“In the last 7 days, I felt unhappy or sad” and “In the last 7 days, I have seen one or more of my friends, or I heard them on the telephone”). The 20-item instrument included three subscales: (a) emotional loneliness, which comprised six items focused on emotional abandonment and missing companionship (e.g., “I experience a general sense of emptiness.”); (b) social loneliness, composed of five items assessing feelings of sociability and of having significant relationships (e.g., “There are many people whom I can count on completely.”); and (c) general loneliness, composed of seven items focused on feelings of isolation (e.g., “I feel isolated from others.”). Due to the widespread and worsened feelings of loneliness caused by the quarantine (1, 5), for the purposes of the present study and in light of the high Pearson's *r* correlations among the ILS factors (0.514, *p* < 0.001 between emotional and social loneliness; 0.792, *p* < 0.001 between emotional and general loneliness; 0.642, *p* < 0.001 between social and general loneliness), a total score was generated that included emotional, social, and general loneliness, which showed an excellent Cronbach's α (0.92).

#### Anxiety Subscale of the Depression-Anxiety-Stress Scale-21

The Anxiety subscale of the Italian version of the Depression-Anxiety-Stress Scale-21 (DASS-21) [(94); for the original English version, see (95)] was used (e.g., “In the last 7 days, I have had breathing problems”). This subscale assesses the frequency and severity of experiencing negative emotions over the previous week on a 4-point Likert scale (from 0 = *did not apply to me at all* to 3 = *applied to me very much, or most of the time*). Cronbach's α value was good (0.86).

#### Bergen Social Media Addiction Scale

The Italian version of the Bergen Social Media Addiction Scale (BSMAS) [(96); original English version by (97)] was used to evaluate problematic social media use (e.g., “How often during the last year have you spent a lot of time thinking about social media or planned use of social media?”). The BSMAS is a six-item scale rated on a 5-point Likert scale ranging from 1 (*very rarely*) to 5 (*very often*), referring to salience, mood modification, tolerance, withdrawal, conflict, and relapse. The Cronbach's α value was 0.80.

Further scales were administered to this sample, but they are not relevant for the current study and will be discussed elsewhere.

### Statistical Analysis

Descriptive statistics were examined for all of the study variables. Multivariate analysis of variance (MANOVA) was used to examine the differences between men and women and between emerging adults (18–35 years) and adults (older than 35 years). Pearson's *r* correlations between the study variables were examined. A mediation model was tested by using Model 4 of Hayes's (98) Process Macro for SPSS, with 1,000 bias-corrected bootstrap samples to test the mediating effect of excessive social media use between participants' perceived loneliness and anxiety.

## Results

### Descriptive Statistics

Among the participants, 55.8% were single, and only 5.9% were living alone during the quarantine. The most used social media were WhatsApp (90.2%), Instagram (64.2%), Facebook (63.6%), Facebook Messenger (16.1%), and Twitter (5.3%). Before the forced isolation due to COVID-19, 39.7% of the participants reported that they spent 1–2 h/day on social media, and only 7.4% spent more than 4 h/day. During the quarantine, the percentage corresponding to 1–2 h/day decreased to 26.7%, and 21.2% of the participants declared that they spent more than 4 h/day on social media. The MANOVA (Table 1) exploring group differences (males/females, and emerging adults/adults) in relation to hours per day spent on social media, loneliness, anxiety, and excessive social media use showed significant differences between gender-based groups [Wilks' λ = 0.98; *F*_(4, 708)_ = 4.5; *p* = 0.001] and age-based groups [Wilks' λ = 0.94; *F*_(4, 708)_ = 10.62; *p* < 0.001]. Bivariate correlations among variables showed a significant co-occurrence of loneliness with all involved variables, especially anxiety (Table 2), with the only exception of gender.

**Table 1:** Means, standard deviations, and comparisons between male/female groups and young adult/adult groups.

	**Total sample**	**Gender**			**Age**		
	***M* (*SD*)**	**Males [*M* (*SD*)]**	**Females [*M* (*SD*)]**	***F*_**(1, 713)**_**	**Young adults [*M* (*SD*)]**	**Adults [*M* (*SD*)]**	***F*_**(1, 713)**_**
Hours/day on social media	3.30 (1.47)	3.13 (1.51)	3.37 (1.45)	3.93^*^	3.51 (146)	2.80 (1.38)	31.45^***^
Loneliness	1.9 (0.58)	1.96 (0.53)	1.88 (0.60)	4.76^*^	1.96 (0.58)	1.76 (0.54)	9.71^**^
Anxiety	0.69 (0.67)	0.59 (0.60)	0.73 (0.69)	4.01^*^	0.76 (0.69)	0.52 (0.56)	13.01^***^
Excessive social media use	2.1 (0.81)	2.12 (0.86)	2.08 (0.79)	0.03^ns^	2.16 (0.80)	1.94 (0.81)	10.51^**^

* p < 0.05;

** p < 0.01;

*** *p < 0.001*.

**Table 2:** Bivariate correlations between all variables estimated with 1,000 bootstrap sample.

	**1**	**2**	**3**	**4**	**5**	**6**	**7**	**8**
1. Gender	–							
2. Age	0.000	–						
3. Marital status	0.080^*^	−0.276^***^	–					
4. Living alone during COVID-19	0.000	0.129^**^	0.137^***^	–				
5. Hours/day on social media	−0.073	−0.220^***^	0.120^**^	0.058	–			
6. Loneliness	0.061	−0.156^***^	0.184^***^	0.082^*^	0.090^*^	–		
7. Anxiety	−0.096^**^	−0.162^***^	0.108^**^	0.014	0.191^***^	0.397^***^	–	
8. Excessive social media use	0.022	−0.119^**^	0.076^*^	−0.054	0.338^***^	0.276^***^	0.331^***^	–

* p < 0.05;

** p < 0.01;

*** *p < 0.001*.

### Mediation Analysis

**Figure 1:**
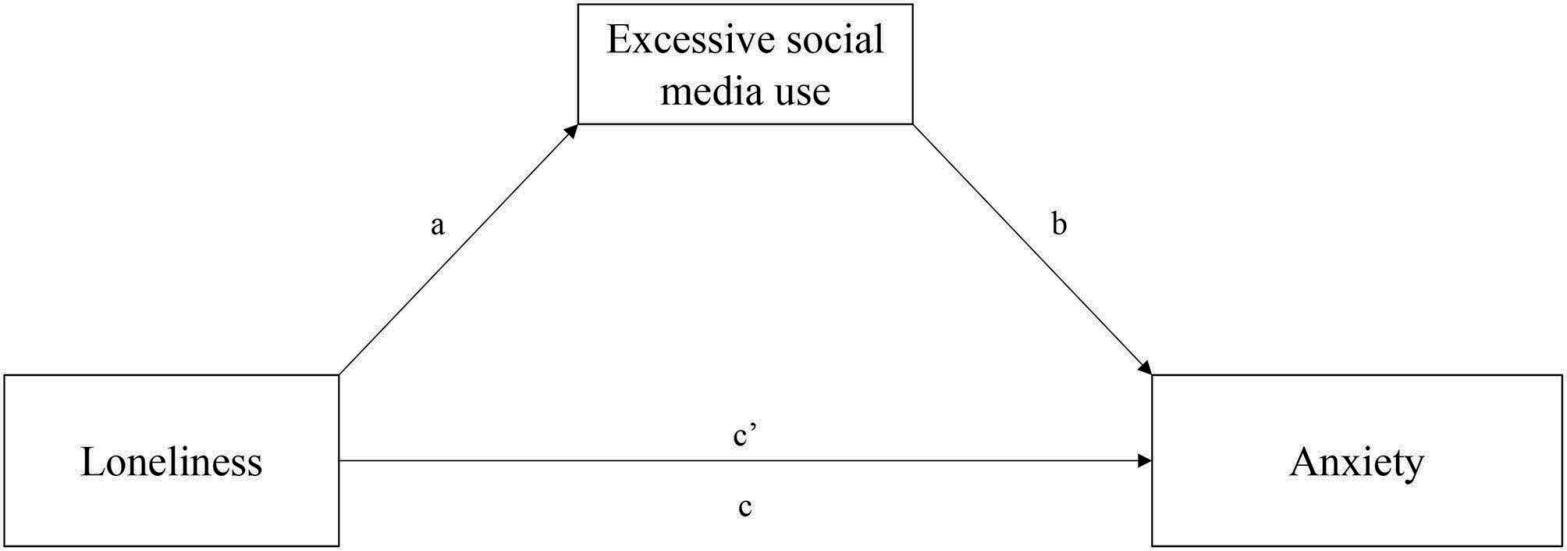
The proposed mediation model.

Concerning the tested mediation model (Figure 1), after controlling for participants' gender (females coded as 0, males coded as 1; β = −0.17; *p* = 0.001), age (β = −0.004; *p* = 0.06, ns), marital status (single coded as 0, in a relationship coded as 1; β = 0.02; *p* = 0.66, ns), living alone during the quarantine (no coded as 0, yes coded as 1; β = −0.04; *p* = 0.68, ns), and hours per day spent on social media (β = 0.06; *p* < 0.001), it confirmed the direct predictive effect of perceived loneliness on anxiety (β = 0.37; *p* < 0.001) and on excessive social media use (β = 0.34; *p* < 0.001), which in turn directly predicted anxiety (β = 0.17; *p* < 0.001). The total effect of perceived loneliness on anxiety was significant (β = 0.43; *p* < 0.001) and the bias-corrected bootstrapping mediation test indicated that loneliness predicted anxiety via excessive social media use (β = 0.06; bootstrap 95%CI [0.03, 0.09]; *p* < 0.001). The Sobel test showed that this model was significant (*Z* = 4.52; *SE* = 0.01; *p* < 0.001), and it explained 23% of the total variance of anxiety (Table 3).

**Table 3:** The effect of perceived loneliness on anxiety with mediating effect of excessive social media use.

		**Model 1 (anxiety)**		**Model 2 (excessive social media use)**		**Model 3 (anxiety)**	
		**β**	***t***	**β**	***t***	**β**	***t***
Loneliness		0.37	9.23^***^	0.34	6.96^***^	0.43	10.8^***^
Excessive social media use						0.17	5.72^***^
*R*^2^	0.23^***^						
*F*_(7, 707)_	30.96						

*** *p < 0.001*.

## Discussion

As many recent studies on the cognitive and emotional effects of COVID-19 pandemic have suggested (2, 4, 6–13, 20), the COVID-19 emergency presents many risks to individuals' mental health. Fear of contamination has led to physical isolation as a global response, impacting on the individual responses to the health crisis (99). This isolation has probably reinforced subjective feelings of loneliness in both older and younger people (1, 5), likely strengthening the individuals' need to be part of virtual communities. In fact, the participants' preference for the use of specific social media and apps for instant messaging was in line with previous findings (100), but in our study people declared they spent more time using social media than before the forced isolation.

In the last several months, social media use has been highly recommended to obtain health and safety information and maintain social contacts in order to face the pandemic's isolation (53). Likely as a result of the distressing situation, social media use has been suggested as a temporary means of recovery from distress and as a coping strategy—which needs to be carefully managed—for facing loneliness and negative emotions (54). In this regard, social media and virtual communities allow users to interact with other people, reinforce relationships, disseminate contents, share common interests, experiences, and emotions [e.g., (101–105)], and also improve their engagement in digital platforms (103, 106, 107). However, social media involvement risks to become excessive or dysfunctional, by triggering a behavior–reward feedback loop (84, 108–110) that reinforces negative moods and supports a vicious use of social media.

In fact, in the compensatory model of problematic Internet-related activities, it is postulated that the reactions to negative life circumstances might lead to excessive Internet use and addictive-like symptoms (92); yet, in such theoretical framework, these symptoms might represent the temporary outcome of a maladaptive and transitory solution to a distressing situation rather than an actual psychopathological condition. This consideration also explains our preference for terms, such as “excessive social media use” and “addictive-like social media use” when discussing a disproportionate engagement in social network, rather than the more common (but often incorrect on the theoretical and clinical level) “social media addiction”: the specific circumstance and reasons for an excessive media use should be carefully examined and addressed before applying the “addiction” label to it (111), and individuals should not be generally and indiscriminately pathologized for their social media use (112), especially during a pandemic isolation (113).

In this sample of Italian adults, the tested mediation model suggested that perceived loneliness during COVID-19 pandemic was positively associated, both directly and indirectly, with anxiety. Furthermore, increased feelings of loneliness and isolation predicted high levels of both anxiety and excessive social media use, in addition, when we controlled for excessive social media use, the predictive effect of loneliness on anxiety further increased. Likely, the facilitated and prolonged access to social media has been a common individual response to stay connected during the quarantine, thus it is possible that people increasingly engaged in social media in an attempt to face their perceived isolation, acting as problematic users in this circumstance. However, this solution may reflect a fear of invisibility and inaction due to the pandemic (3) that has proved to be unsuccessful for lonelier people, whose feelings of anxiety increased. Likely, even though online social interactions can act as a temporary useful solution that allows individuals to keep in touch with other people, thus fostering social support (54) and allowing individuals to feel less alone (1), it seems that in the medium to long run, online social contacts cannot substitute offline social interactions in reducing feelings of loneliness and anxiety (84). Indeed, research shows that online social interactions tend to enhance well-being, social belonging, and relationship quality when used in combination with offline social interactions (85, 114, 115). Thus, in line with theory (116), online interactions do not provide a definitive solution to relieve users from their subjective sense of isolation during a prolonged absence of further social contacts outside the household. Overall, our findings suggest that exclusive and excessive use of social media has likely acted as a coping strategy for individuals' feelings of loneliness. However, in some cases, this may represent a maladaptive strategy that might foster a dysfunctional feedback loop reinforcing lonely individuals' anxiety in the specific pandemic circumstance. Accordingly, problematic social media use has already been evidenced as a dysfunctional emotional-regulation strategy (117–119), although it is frequently used to control mood (120–124). Thus, despite this excessive social media use denoting individuals' efforts to face their sense of loneliness and isolation, it might also foster more negative outcomes if forced by the situation and prolonged in time.

Evidence also showed that the increase in negative feelings was stronger among women and younger participants when examining the effects of sociodemographic variables in the mediation models, which is in line with vast amounts of literature suggesting increased internalizing symptoms in females (125), even during the pandemic crisis (12) and greater difficulties with emotional regulation among younger people (126). Moreover, according to literature, in this sample, women and younger adults seem to not only be more engaged in online social connections (71, 85) but also more exposed to negative moods. Finally, they seem to use social media more dysfunctionally for controlling their feelings of loneliness, and this might have reinforced their feelings of anxiety.

This study has some limitations that need to be addressed. First, the cross-sectional design limited the ability to formally test causative effects. Second, despite the participants coming from the entire Italian peninsula, the different geographic areas of Italy have been differently affected by the COVID-19-related health crisis, limiting the generalizability of results. Moreover, the present study explored only a small number of variables in relation to the complexity of the relationship among feelings of loneliness, excessive social media use, and anxiety during the COVID-19 pandemic. In particular, information about the participants' general health status during the pandemic and the presence of direct or indirect contact with the virus has not been collected in our study. Furthermore, it has been demonstrated that individuals' temperament and characteristics in the psychological response to the ongoing pandemic provide insights into developing tailored intervention strategies and need to be better investigated (99). Future research may examine the role of such variables to improve our understanding of individuals' social media use during the pandemic and to further identify specific groups of people that might be more vulnerable to problematic use. Finally, this study evaluated general use of social media during the pandemic, including apps for instant messaging. Further research could explore whether the use of specific social media is related with an excessive involvement and addictive-like symptoms.

These limitations notwithstanding, our findings suggest that excessive social media use was associated with increased feelings of loneliness and anxiety during the COVID-19 pandemic. The current pandemic is not only changing priorities for the general population but it is also challenging the agenda of health professionals, including that of psychiatrists and other mental health professionals Accordingly, clinical interventions with people who will continue to display excessive social media use after the pandemic resolution might specifically address their feelings of loneliness that may prompt such dysfunctional use and foster anxiety. Moreover, clinicians might successfully orient problem-focused coping styles toward helping people facing loneliness (127). Additionally, preventative actions need to be taken to improve literacy about media consumption among the general population, to help individuals to adequately use social media, and to avoid the risks associated with excessive social media use during pandemics.

Furthermore, mental health clinicians need to be directly involved in the management of the crisis and post-crisis also as part of policy task forces (20), since the ongoing as well as the lasting effects of the pandemic on individuals' behaviors need to be accounted for. Indeed, lonely people are already at risk of preferring online social interactions (78, 85), which displace time spent in offline social activities. The global epidemic has probably exponentially enlarged the number of lonely individuals, suggesting that their long-lasting use of social platforms must be addressed. The risky impacts of over or even exclusive involvement in online activities on people restarting their offline lives and relationships after the COVID-19 emergency deserves particular attention, considering the potential danger of prolonged psychological and socioemotional withdrawal when the pandemic ends. For this reason, longitudinal designs are greatly needed to analyze the pandemic's effects on social media use in different populations more in greater depth, and the differences and similarities between different cultural contexts should be explored. Yet, the findings of this study already suggest that clinicians should carefully assess and eventually treat feelings of loneliness and internalizing symptoms, such as fear and anxiety due to the COVID-19 in the post-pandemic world (12) and support the view that boosting literacy about social media use across the population could be critical to promote adaptive alternatives for socialization without fostering maladaptive involvements in the digital world.

## Data Availability Statement

The raw data supporting the conclusions of this article will be made available by the authors, without undue reservation.

## Ethics Statement

The studies involving human participants were reviewed and approved by Ethical Committee of Psychological Research Department of Humanities—University of Naples Federico II. The patients/participants provided their written informed consent to participate in this study.

## Author Contributions

VB designed the study, led the literature research, and wrote the manuscript. FG contributed to data collection and led the statistical analysis. AM conceptually contributed to the development of the work and edited the manuscript. AS critically revised the whole work for important intellectual content and edited the manuscript. All authors read and approved the final version of the paper.

## Conflict of Interest

The authors declare that the research was conducted in the absence of any commercial or financial relationships that could be construed as a potential conflict of interest.
